# Germany’s first COVID-19 deceased: a 59-year-old man presenting with diffuse alveolar damage due to SARS-CoV-2 infection

**DOI:** 10.1007/s00428-020-02872-y

**Published:** 2020-07-04

**Authors:** Fabian Heinrich, Jan-Peter Sperhake, Axel Heinemann, Herbert Mushumba, Maximilian Lennartz, Dominik Nörz, Markus Glatzel, Marc Lütgehetmann, Klaus Püschel

**Affiliations:** 1 Institute of Legal Medicine, University Medical Center Hamburg-Eppendorf, Butenfeld 34, 22529 Hamburg, Germany; 2 Institute of Pathology, University Medical Center Hamburg-Eppendorf, Hamburg, Germany; 3 Institute of Medical Microbiology, Virology and Hygiene, University Medical Center Hamburg-Eppendorf, Hamburg, Germany; 4 Institute of Neuropathology, University Medical Center Hamburg-Eppendorf, Hamburg, Germany

**Keywords:** COVID-19, Autopsy, Micro-thrombosis, SARS-CoV-2, Morphology

## Abstract

Clinical characterization of COVID-19 (Corona Virus Disease 2019) is being performed worldwide to gain insights into the pathogenesis and course of the disease. Little is known regarding morphological findings, which are essential to understanding the unique features and pathomechanisms of the disease, from which one can identify possible new treatments. It has been shown that diffuse alveolar damage, signifying acute respiratory distress syndrome, is present together with atypical multinucleated cells in reported cases of the disease by Tian et al. (J Thorac Oncol 15:700–704, 2020). Macroscopic morphological findings in COVID-19 remain elusive to this day. Here, we report the case of the first German to die due to COVID-19. A detailed examination consisting of full-body computed tomography, autopsy, histology assessment, and viral assessment has been performed. The lungs of the deceased contained high concentrations of SARS-CoV-2 RNA and displayed the typical radiological signatures of COVID-19. Furthermore, a morphological pattern was found displaying hyperaemic areas interspersed by normally perfused areas affecting the whole lung. We also report a finding suggestive of micro-thrombotic events in the lung, which is compatible with the recently described coagulation changes and increased incidence of pulmonary artery embolisms seen in COVID-19 patients as reported by Wichmann et al. (Ann Intern Med, 2020). A broader study is needed to confirm these findings.

**Electronic supplementary material:**

The online version of this article (10.1007/s00428-020-02872-y) contains supplementary material, which is available to authorized users.

## Introduction

SARS-CoV-2 first attracted attention in the Wuhan region of China in December 2019 through a cluster of pneumonia of unknown origin [1]. The World Health Organization has since declared the global distribution of SARS-CoV-2 to be a pandemic [2]. The numbers of new infections and deaths are constantly rising, which demonstrates the urgent need for detailed investigations into COVID-19 [3].

Heshui Shi et al. recently described one of the dominant features of COVID-19 pneumonia in CT diagnostics to be peripheral ground-glass opacification involving both lungs [4]. The histopathological features of COVID-19 in patients with lung resection due to adenocarcinoma have revealed diffuse alveolar damage with pneumocyte activation and inflammatory infiltrates [5, 6]. Systematic reports on morphological findings are still missing. Lui Xi and colleagues have published a case report using a minimally invasive autopsy technique, in which they describe macroscopic dark-red discoloration of both lungs interspersed by greyish-white lesions [4].

Here we report the systematic examination, including postmortem CT scan, autopsy, histology, and virology assessments, of the first German to die from COVID-19.

## Methods

### External/internal examination

Internal and external examination was performed according to the S1 guidelines “Rules for conducting a medical autopsy” and “The forensic postmortem examination” of the German Society of Legal Medicine, with consideration of the recommendations on HG3-related work [7]. Detailed written and photographic documentation was performed to record the findings.

### Computed tomography

A computed-tomography examination has been performed using a Philips Brilliance 16-slice MDCT. Whole-body CT were performed from top to thigh (slice thickness 1 mm, Pitch 1.5, 120 kV, 230–250 mAs), complemented by dedicated scans of the thorax with higher resolution (slice thickness 0.8 mm, Pitch 1.0, 120 kV, 230–250 mAs).

### Histology

Histology samples of the brain, pharyngeal/tracheal mucosa, lungs, heart, kidney, liver, spleen, small intestines, and testicles were processed. The tissues were fixed in formalin with extended fixation times of up to 1 week and subsequently embedded in paraffin. Staining was performed using H&E staining and immunohistochemistry for CD8^+^ cells (αCD8 monoclonal Antibody, 1:100, clone SP239, Spring Bioscience, Pleasanton, USA).

### Virology

Quantitative SARS-CoV-2 RNA PCR was performed with tissue samples from the brain, pharyngeal-/tracheal mucosa, lungs, heart, kidney, liver, spleen, small intestines, and testicles. Furthermore, bodily fluids such as urine, feces, blood, and cerebrospinal fluid were analyzed. Automatic nucleic acid extraction followed by one-step rt-PCR was performed using a LightCycler480 system (Roche). Ct values for the target SARS-CoV-2 RNA (FAM) were determined. For quantification, standard in vitro-transcribed RNA of the E gene of SARS-CoV-2 was used [8]. Samples revealing Ct values above 35 were defined as negative.

## Results

### Clinical course

One month prior to death, the patient went on a cruise to Hurghada, Egypt. One day after his arrival, he developed mild general symptoms. Dyspnea occurred 8 days later. Shortly afterwards, he was admitted to hospital, and during the first night developed a fever and productive cough. After 6 days in hospital, the patient passed away. The immediate cause of death was reported as severe respiratory failure due to COVID-19. Detailed clinical data is unavailable. The deceased was transported to Hamburg, Germany, within 12 days of death and systematically examined at the Department of Legal Medicine. The patient’s risk constellation for COVID-19 exposure has been assessed and revealed a positive-index patient who had been on the cruise ship and subsequently died due to COVID-19.

### External examination

The 59-year-old patient presented 12 days postmortem embalmed with formalin and with subtle signs of decomposition. The patient was in good care condition with signs of standard medical care such as peripheral vascular catheters. He was obese (BMI 32.8 kg/m^2^) but exhibited no other externally visible signs of pre-existing diseases. At first glance, general signs of cyanosis, such as lividity of the skin, were visible. The medical records solely report medicated arterial hypertension.

### Computed tomography

The radiological assessment with computed tomography examination of the deceased showed moderate bilateral pleura effusions. Ground-glass opacifications in sub-pleural areas are noted. Furthermore, attenuations converging towards the center of the lung resembling ground-glass density nodules can be seen. Global multifocal reticular consolidation was found, with accentuation in the central areas of both lungs. Enhanced consolidation at the dorsal boundaries and maintained air space restriction in the apical-ventral lung areas are consistent with postmortem hypostasis. Significant crazy-paving patterns could not be identified (Online Resource 1).

### Internal examination

**Fig. 1:**
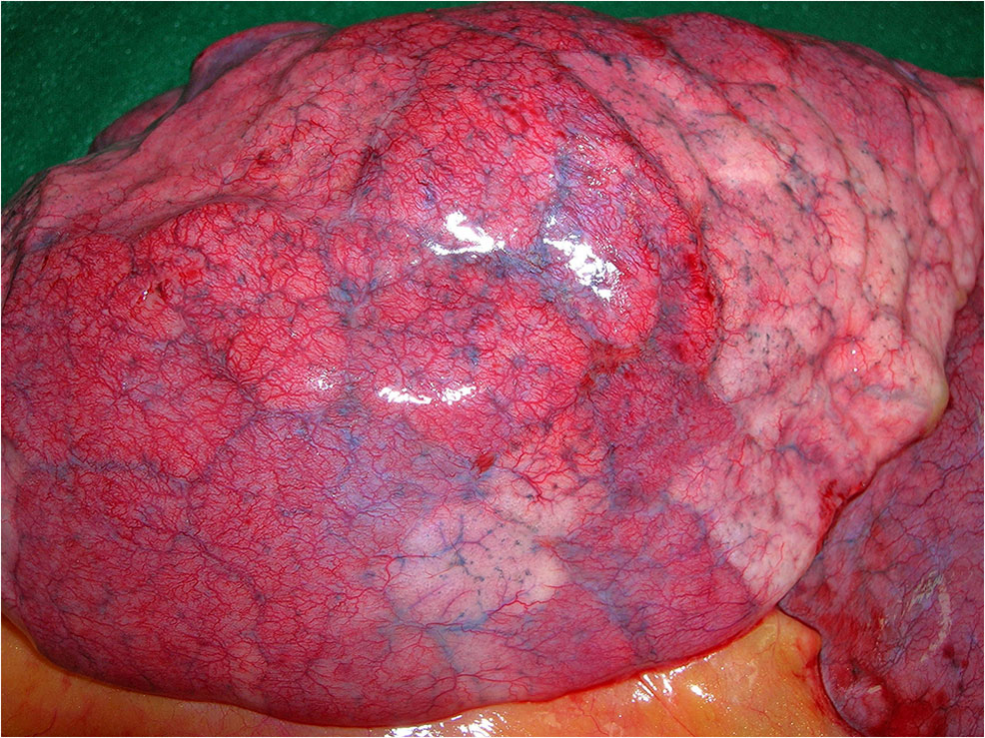
Deep-red, hyperaemic discolorations of the right lung surface interspersed by paler grayish-white areas

An internal examination of the three body cavities was performed. Distended and heavy lungs with a weight of 820 (left) and 980 g (right) were visible. In accordance with lung edema, foamy hemorrhagic fluid was found up to the upper respiratory tract. Strikingly, a morphologic pattern with deep-red discolorations presented in the whole lung. The affected areas were slightly nodular, dense, and hyperaemic in comparison with paler normal areas and evenly distributed in large patches over the surfaces of the lungs (Fig. 1). An involvement of the trachea with signs of both acute hemorrhagic tracheitis and bronchitis with patchy mucosal bleeding, without visible bacterial impairment, accompanied the finding. Incidentally, we found signs of a congestive cardiomyopathy (dilated atria and ventricles), extensive lipomatosis cordis with a heart weight of 600 g, and moderate arteriosclerosis without signs of previous myocardial infarctions. The abdominal organs exhibited signs of congestion with minimal signs of hepatomegaly (1800 g) and hepatic steatosis. Multiple renal cysts up to 2-cm large were noted. Apart from minimal signs of arteriosclerosis of the cerebral arteries, the brain exhibited no pathological signs macroscopically. No other organs presented abnormalities.

### Histology

**Fig. 2:**
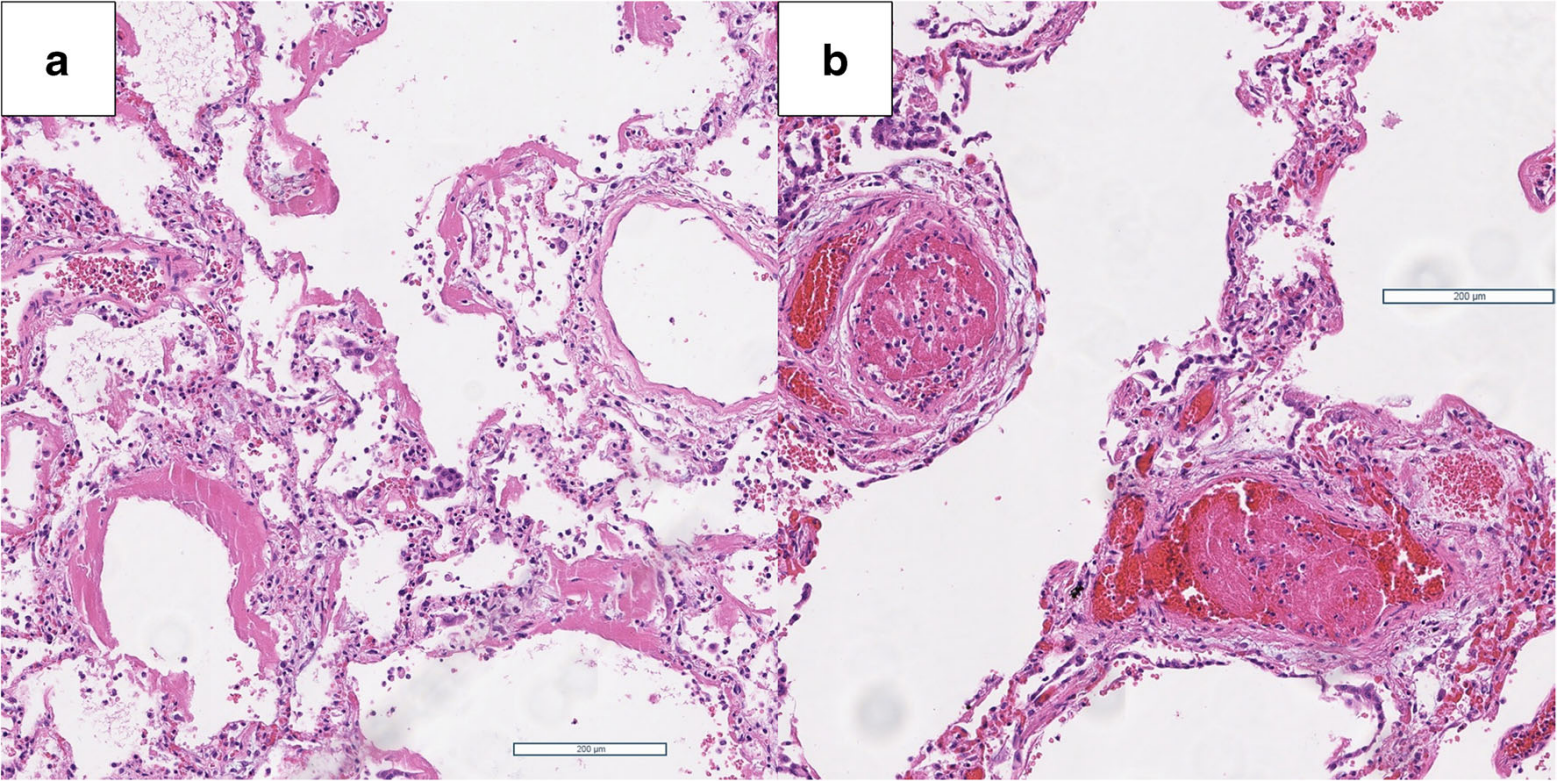
**a** Prominent hyaline membranes, moderate infiltration of mononuclear inflammatory cells (H&E, × 50) accompanied by **b** microvascular thrombemboli (H&E, × 10)

Histopathology of the lungs revealed diffuse alveolar damage (Fig. 2a). Ubiquity of prominent hyaline membranes accompanied by microvascular thrombemboli (Fig. 2b), capillary congestion, and protein-enriched interstitial and intra-alveolar edema dominated the picture. Moderate infiltration of mononuclear inflammatory cells was present, predominantly constituting lymphocytes in the absence of granulocytes. Pneumocytes in areas with prominent hyaline membranes were hyperplastic, indicating focal activation. Focal multinucleated syncytial cells infiltrated the alveoli. Except for these, no other pathological features in the lungs, such as vasculitis or endothelitis, were visible. The heart showed signs of advanced interstitial and perivascular myocardial fibrosis and biventricular lipomatosis. The abdominal organs apart from the liver, revealing signs of unspecific lymphoplasmacellular hepatitis and centro-lobular lipomatosis, showed no pathological signs. The neuropathological examination showed signs of a brain-stem pronounced unspecific immune response with perivascular and parenchymal infiltration of CD8^+^ cells (Online Resource 2) and minimal signs of arteriosclerosis of the cerebral arteries.

### Molecular genetic analysis

Postmortem molecular genetic testing in combined nasopharyngeal and oropharyngeal swab diagnostics showed a highly positive nucleic acid test for SARS-CoV-2 with 1.2 × 10^6^ viral copies, confirming the suspected SARS-CoV-2 infection. We examined different organs for the prevalence of viral components and found unsurprisingly high viral loads in the lung with up to 6.34 × 10^6^ viral copies. Other organs screened showed no evidence for SARS-CoV-2 RNA. Screened bodily fluids also exhibited no evidence for SARS-CoV-2 RNA (Table 1).

**Table 1:** Quantitative SARS-CoV-2 RNA PCR from different tissues

Tissue	Ct value^(^*^)^	SARS-COV-2 RNA copies/ml
Pharyngeal swab	27.0	1.2 × 10^6^
Lung	24.6	6.3 × 10^6^
Cerebral cortex	-	Negative
Heart	-	Negative
Liver	-	Negative
Kidney	-	Negative
Spleen	-	Negative
Small intestine	-	Negative
Testicles	-	Negative
Blood	-	Negative
Cerebrospinal fluid	-	Negative
Feces	-	Negative
Urine	-	Negative

^(^*^)^Values below the cut-off (Ct < 35) are indicated by “-”

## Discussion

Here, we report the case of the first German COVID-19 deceased—a 59-year-old male. The COVID-19 infection was presumably acquired during a cruise ship tour to Hurghada, Egypt, and initial general symptoms deteriorated into respiratory symptoms with cough and dyspnea. After a short clinical course, the patient died in Egypt and was subsequently autopsied at the Department of Legal Medicine, UKE Hamburg.

CT examination revealed ground-glass opacifications, especially in the sub-pleural areas, as previously described in other COVID-19 patients [9]. Furthermore, pleural effusions, which are not typically observed in COVID-19 patients, were found in the postmortem CT scan. These might be an artifact due to increased postmortem intervals or embalming procedures [10]. Detailed information on how the embalming procedures in Egypt were performed are missing; however, it is possible that the lungs might have been affected by these procedures.

The lungs exhibit macroscopic deep-red discolorations interspersed by paler areas (Fig. 1). The morphological pattern of hyperaemically and normally perfused areas within the lung is compatible with micro-thrombotic events. This might explain the coagulopathy-associated mortality in COVID-19 patients as recently shown by Zhou et al. [11]. Increased incidence of thrombotic events and arterial-pulmonary embolisms as reported by Wichmann et al. supports this hypothesis [12]. The cardiomegaly and hepatomegaly seen on autopsy were deemed to be significant, although a clinical examination 3 months prior to death had shown no abnormalities.

Histopathologic results showed diffuse alveolar damage consistent with early acute respiratory distress syndrome. The predominant findings are in alignment with recently described early histopathologic changes in an early course of COVID-19 [6]. Moderate degree of inflammatory infiltrate concurs with clinically described leukopenia in COVID-19 patients [9]. The strong presence of lymphocytes in the infiltration fits the picture of a viral pathogenesis. Recently, meningoencephalitis due to SARS-CoV-2 has been described [13]. We did not observe overt signs of encephalitis but rather a brain-stem dominant immunological reaction which indicates the need for further examinations.

Molecular analysis confirmed the clinical diagnosis of COVID-19 with high viral RNA copy numbers in the pharynx and the lungs. No SARS-CoV-2 RNA was detected in bodily fluids or the other organs. However, the procedure of embalming with formalin containing fluids and a long postmortem interval might lead to RNA degradation and false negative results of the qPCR [14].

To summarize, the patient suffered a severe course of SARS-CoV-2 infection. Performing systematic examination, we revealed a macroscopic pattern for COVID-19-related changes, which is most likely explained by (micro-) thrombotic events. Given that only one case is presented here, further systematic examination of deceased COVID-19 patients is needed.

## Electronic supplementary material

ESM 1 Axial (a) and transversal (b) chest computed tomography scan (120 kV, 230-250mAS) revealing ground-glass opacifications in sub-pleural areas with converging attenuations resembling ground-glass density nodules. Global multifocal reticular consolidation with prominence in the central areas of both lungs (JPG 1711 kb)

ESM 2 Section of the upper medulla oblongata (H&E, × 25) (a) exhibits unspecific immune reaction with perivascular and parenchymal infiltration of CD8^+^ cells (CD8 immunostaining, × 25) (b) (JPG 3484 kb)
