# Supplementary figures and images for: Germany’s first COVID-19 deceased: a 59-year-old man presenting with diffuse alveolar damage due to SARS-CoV-2 infection
Source: Virchows Arch. 2020 Jul 4;477(3):335–9. doi: 10.1007/s00428-020-02872-y (PMC7359760; doi:10.1007/s00428-020-02872-y)

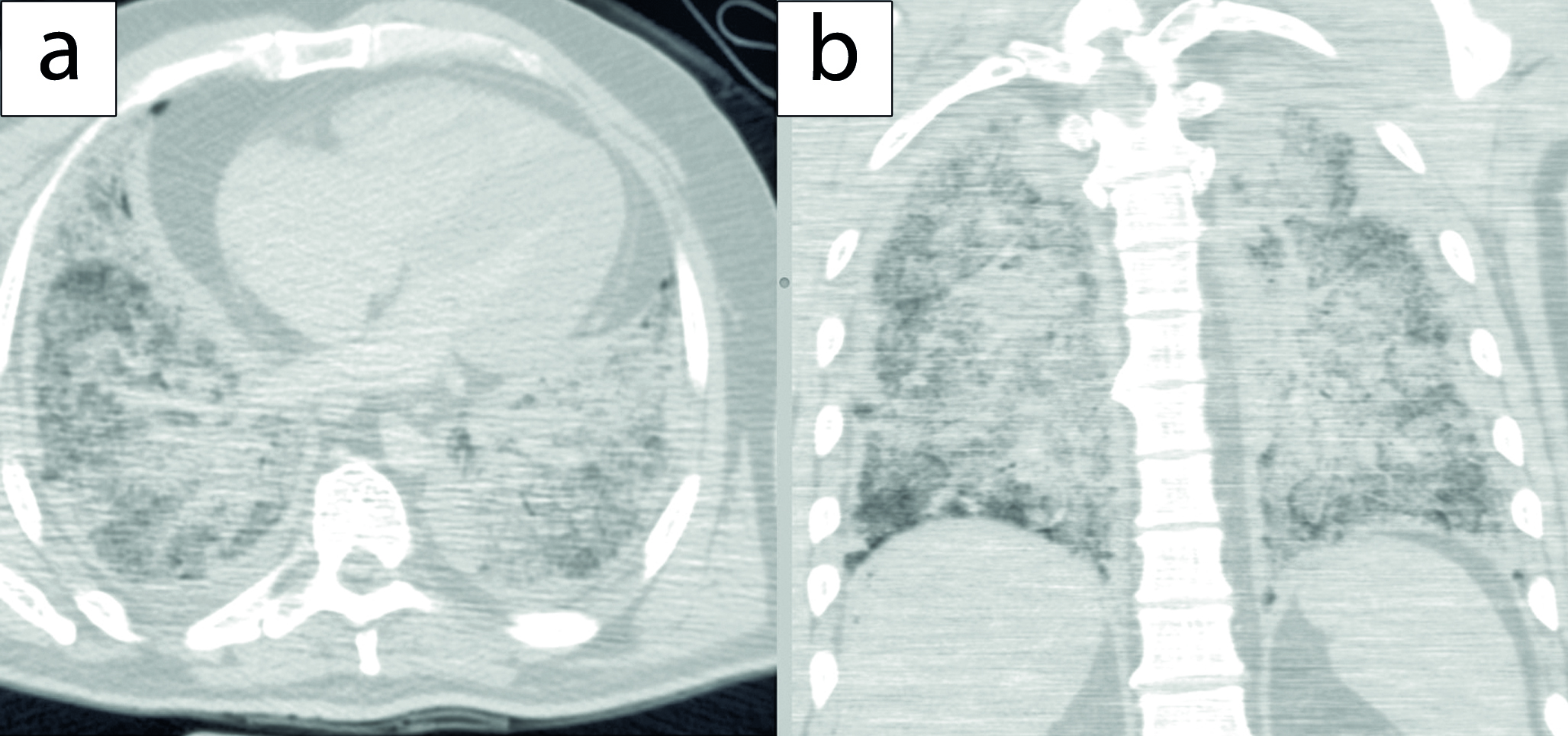

Supplement: Supplementary file 1 — Axial (a) and transversal (b) chest computed tomography scan (120 kV, 230-250mAS) revealing ground-glass opacifications in sub-pleural areas with converging attenuations resembling ground-glass density nodules. Global multifocal reticular consolidation with prominence in the central areas of both lungs (JPG 1711 kb) [file 428_2020_2872_MOESM1_ESM.jpg]

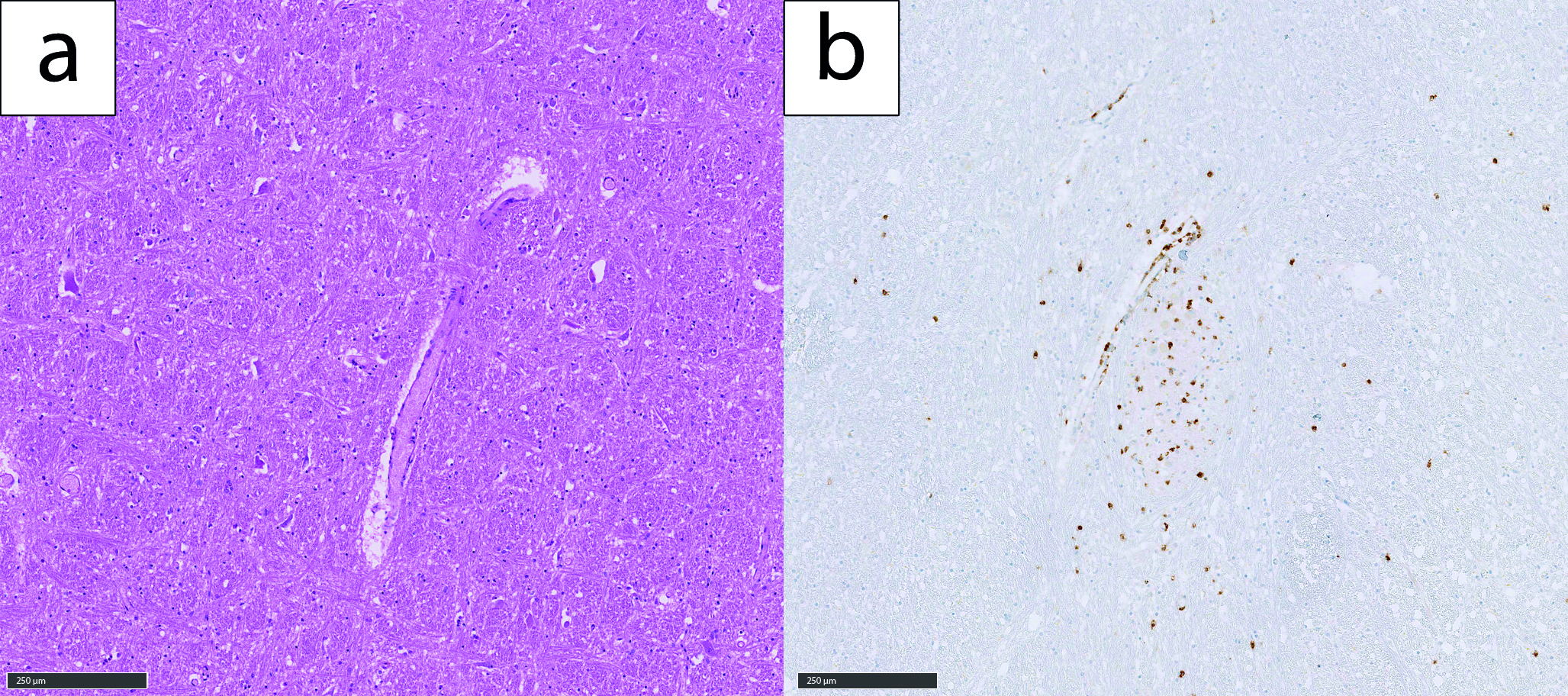

Supplement: Supplementary file 2 — Section of the upper medulla oblongata (H&E, × 25) (a) exhibits unspecific immune reaction with perivascular and parenchymal infiltration of CD8+ cells (CD8 immunostaining, × 25) (b) (JPG 3484 kb) [file 428_2020_2872_MOESM2_ESM.jpg]
